# A novel approximation of underwater robotic vehicle controller exploiting multi-point matching

**DOI:** 10.1038/s41598-025-14612-w

**Published:** 2025-08-22

**Authors:** Umesh Kumar Yadav, V. P. Singh, Luigi Fortuna, Umesh Kumar Sahu

**Affiliations:** 1 Department of Electrical Engineering, Malaviya National Institute of Technology, Jaipur, 302017 India; 2 Department of Electrical Electronic and Computer Engineering, University of Catania, 95124 Catania, Italy; 3 CNR-IASI, Italian National Research Council, Institute for Systems Analysis and Computer Science, 00185 Rome, Italy; 4 Department of Mechatronics, Manipal Institute of Technology, Manipal Academy of Higher Education, Manipal, Karnataka 576104 India

**Keywords:** Approximation, Expansion-parameters, Greywolf optimization algorithm, Lower-order model, Multi-point matching, Underwater robotic vehicle controller., Electrical and electronic engineering, Engineering

## Abstract

This proposed work is presenting the approximation of higher-order (HO) underwater robotic vehicle (URV) controller with the help of multi-point matching technique by incorporating greywolf optimization algorithm (GWOA). The performance of URV system is affected by external and internal dynamics. The proper momentum of URV system is achieved by designing a controller. The URV can be effectively operated by control action of controller. The URV controller is approximated to comparatively lower-order (LO) to propose an efficient, effective and economical controller for HOURV system. The approximation is accomplished with the help of expansion parameters of HOURV controller and its desired LOURV controller. The errors between these expansion parameters of HOURV controller and its desired LOURV controller are minimized using multi-point matching. The multi-point matching is depicted in the form of objective function (OF). The constructed OF is minimized by exploiting GWOA by fulfilling the steady-state matching condition and Hurwitz stability criterion, as constraints. The effectiveness of proposed approach of multi-point matching is verified by comparing the proposed LOURV model with LOURV models obtained with the help of other approximation approaches. The applicability of proposed LOURV controller is evaluated and validated by analyzing responses and tabulated data obtained in the results. Additionally, the statistical data of performance error values (PEVs) are provided in tabulated form along with its bar plot.

## Introduction

In this modern era, due to technological development, learning based approaches are employed in several domestic, engineering, industrial, biomedical, aerospace, security, etc. fields with the help of artificial intelligence and machine learning based tools^1,2^. In these areas, robotic is evolved as omniscient and premier branch of science. Robotics is one of the most significant and important field of research due to its usability and wide applications^3^.

In the momentum based robot design, robots are classified as linear momentum based robot and axial momentum based robot^4^. These robots can be utilized in ground applications, over-ground applications, surface-water applications, underwater applications, etc.^5^. In these applications, most of the robotic systems are deployed as non-holonomic robotic systems^6^. In robotic vehicles, the number of dependent states and its degree-of-freedom are different due to non-holonomicity of robotic systems. In case of underwater and floating type robotic systems^7^, the degree-of-freedom and dynamics are different due presence of various state variables. Due to which the analytical approach and mathematical modelling applied for underwater and floating type robotic systems are divergent in comparison to grounded and aerial robotic systems.

In^8^, Kadam and Tiwari designed the controller for autonomous underwater robotic vehicle (URV). In this article^8^, fourth order transfer function in terms of depth with respect to displacement is manifested, and its controller is proposed. Since, the manifested URV transfer function is affected by time-lag due to uncertainty, the linearized third order URV transfer function is obtained and control design is presented for URV system in^8^. The effective and comparatively more efficient controller design can be employed by proposing the comparatively lower-order (LO) URV controller for higher-order (HO) linearized URV system. So, the linearized third order URV controller transfer function can be further utilized for approximation in to desired order of reduced order model. However, the controller design for comparatively lesser order system is quite easier and economical, since the several advantages and merits are associated with reduced order models^9^. The advantages of obtaining LO model for HO system are as follows: analytical and mathematical designing of controller becomes easier, complexity in obtaining control laws becomes lesser, inversion of system matrices in the presence of system dynamics is simpler, economical due to less complication in control design, comparatively minimal simulation time, etc. In general, several order reduction approaches of obtaining LO models are available as Padè approximation, stability equation based reduction, Routh approximation, factor division reduction, etc., for fixed coefficient systems and interval modelled systems. In literature^10^, by addressing system uncertainties and external disturbances effectively through adaptive neural networks and layered control strategies is also presented as approximation strategy. In^11^, the authors extended the concept to HO uncertain nonlinear systems, incorporating active disturbance rejection to improve robustness and transient performance. Some of the other approximation approaches, generally utilized by researchers are Markov parameter and time-moment matching based approach, eigen vector based state space approximation, parametric error minimization based approach, uncertain nonlinear and time-varying system approximation with active disturbance rejection^12^, truncation based method, Hankel’s norm $(H_\infty )$ based approach, multi-point matching based approach, data-driven based reduction, etc. In literature, the described approximation approaches are exploited in the attainment of comparatively LO model for HO system in several applications such as aerospace application^13^, bio-medical application^14^, converter design application^15^, systems engineering application^16^, robotics application^17^, etc.

In this presented work, LO model for HOURV controller is obtained with the help of minimization of errors between expansion parameters of HOURV controller and its desired LOURV model. To perform the error minimization, objective function (OF) is constructed. The OF is a function of expansion parameters of HOURV system and LOURV model. The desired expansion parameters are selected such that the response of LOURV model must be matched with the response of HOURV system in terms of steady state and transient responses. The better approximated LOURV model of order two is obtained by constructing factorized augmented transfer functions (FATFs) using multi-point matching^18^. The detailed explanation and mathematical approach of multi-point matching is presented in problem formulation section. The determination of desired LOURV model of second order is accomplished by incorporating the greywolf optimization algorithm (GWOA) in the framed OF. The minimization using GWOA is fulfilled by satisfying steady-state (SS) matching condition and achieving Hurwitz criterion of stability (HCS) as constraints related to OF. The graphical depiction of obtained results for LOURV models with respect to HOURV system are provided in the form of step, error, impulse and Bode responses. The validation of findings is done by presenting the comparative analysis in terms of tabulated data as time domain specifications and error values with respect to HOURV system in comparison to distinguished LO models obtained by employing other reduction approaches. Moreover, the highlights of the presented work as a prime contribution are as follows:

- LO model for a HOURV controller is obtained by employing multi-point matching method.
- In multi-point matching, FATFs are obtained using coefficients of HOURV controller and its desired LOURV model for minimization of errors.
- To perform error minimization, an OF is constructed with the help of expansion parameters of HOURV controller system and its LOURV model.
- The desired expansion parameters are selected such that the response of LOURV model must be matched with the response of HOURV controller system in terms of steady state and transient responses.
- The determination of desired LOURV model of order two is done by minimizing the framed OF with the help of GWOA.
- Minimization of OF is fulfilled by satisfying constraints of zero SS error and HCS.
- For result validation of proposed LOURV model, comparative analysis is also done with the models ascertained by other approximation methods such as differentiation approximation^19^, factor division approximation^20^, mixed approximation methods^21^ by incorporating Routh approximation, direct truncation, etc.

This proposal is presented by bifurcating the proposed work in different sections: the available literature on URV system and their control design are discussed in section “Related work” as a literature review. Further, in section “Mathematical modelling of underwater robotic vehicle controller”, mathematical modelling by incorporating state space matrices of linearized URV controller is presented. Furthermore, methodology of reduction of HO system to LO model is discussed in section “Problem formulation” as problem formulation. Additionally, minimization approach using GWOA is demonstrated in section “Greywolf optimization algorithm”. Moreover, in section “Numerical solution: approximation of URV controller, results and discussion”, HOURV controller is approximated to second order URV model. This section is also presenting the obtained results and its discussion. Finally, in section “Conclusion”, presented work is concluded on the basis of findings.

## Related work

The conceptualization and implementation of underwater robotic vehicle (URV) by employing mathematical modelling and designing are evolved on the basis of utility, design criterion, area of application, vehicle dynamics, control design, economic design and operation, etc. In general, the URV are classified as single-body type URV and multi-body type URV based on vehicle utility^22^. Some of the classification are done by concerning the design criterion as bio-mimetic shaped, glider shaped, torpedo shaped, etc.^23^. However, the area of applications of URV in surface-water, under-water, submerged applications, URVs are also categorized based on distance from the surface water i.e. heave level^24^. The bifurcation of URV is also employed by considering their mode of operation. In this category, autonomous type URV and remote operated URV are classified^25^, generally. In literature, special types of URVs are also designed as URV-TWINBOT^26^ (TWINBOT stands for twin robot which is utilized for co-operative underwater intervention), URV-COBOTS (COBOTs signifies collaborative-robots)^27^, etc.

Some of the researchers exploited their work by presenting a survey on various types of URV designs and their recent developments. In^28^, Wang et al. presented a comprehensive summary on bio-mimetic types URV and their distinguish control strategies, in details. Similar review is also done in^29^ by Connor et al. by incorporating detailed analysis on sensors and communication technologies involved in the operation of swarm based URV systems. Later, detailed analysis on URV sensors and URV communication technologies is also provided in^30^ and^31^ by Cong et al., and Hoeher et al., respectively. Aditionally, specifically designed underwater robotic system such as multi-robotic URV, underwater gliding type robots, exo-skeleton type wearable robots for different underwater marine applications are also available in the literature^32^. Moreover, the recent developments in URV, different applications of URV, issues and challenges associated with URV are discussed in details by Wibisono et al. in^33^.

In URV, the issues and challenges related to linear and axial momentum control^34^, trajectory tracking, hurdle clearance, hydrodynamic forces, buoyancy effect, vehicle dynamics, etc. can be overcome by proposing a comparatively better controller for URV body. The appropriate control design is one of the prime criterion for accurate and precise operation of URV. The design and control of underwater robot for specific applications have been explored by various researchers in literature. In^35^, several technological developments on URV are presented by discussing their types, applications, design and control, etc. for autonomous mode. The navigation control and robust motion control of URV are discussed by Shi et al., and control design approach are presented by Liu et al., respectively in^36^ and^37^. Similarly, in^38^, Bejarbaneh et al. presented the robust control design for autonomous URV using linear-matrix-inequality exploiting particle-swarm optimization.

Some of the researchers presented their work on trajectory tracking of autonomous URV. In^39^, Guerrero et al. presented the control design strategy based on adaptive disturbance observer for autonomous URV trajectory control. Further, Manzanilla et al. discussed and presented, integral sliding mode control based trajectory control for URV, in^40^. Furthermore, in^41^ and^42^, the tracking control scheme using observer based barrier Lyapunov functions, and sensor based detection of coverage degree with target identification are deployed, respectively. In^43^, Heshmati-alamdari et al. proposed the nonlinear model predictive control scheme (NMPCS) for position control of URV system. The robust stability analysis is also performed for under-actuated URV system by incorporating experimental results in^43^. Similarly, NMPCS is also utilized in^44^ for trajectory tracking of URV manipulator system. In addition, trajectory tracking of URV system is employed by incorporating active fault tolerant control scheme based on adaptive interval observer by Wang in^45^.

In literature numerous control design methods^46^ are adapted by researchers to demonstrate the significance of control dynamics, degree-of-freedom, momentum, etc. of URV for different applications. Since, in autonomous URVs, the control design is most important to balance the degree-of-freedom of vehicle body, some of the researchers exploited their work on analysis and design of control dynamics of URV body by incorporating fuzzy and machine learning based approaches. In^47^, Liu et al. discussed and presented the control strategy using fuzzy approach for trajectory identification. In similar fashion, machine learning based double deep Q-network assisted deep deterministic policy gradient algorithms are utilized for trajectory tracking and phase shift design of URV exploiting re-configurable intelligent surface technique in^48^ by Mei et al. Further, in^49^, fuzzy based motion and trajectory control is implemented for specially designed intelligent ocean-I (IO-I) prototype URV system by Kong et al. Similar type of fuzzy based adaptive controller is also utilized for trajectory control of URV system in^50^ by Lakhekar et al. Furthermore, implementation of adaptive trajectory tracking control is done by Wang et al. in^51^ for linear and angular momentum control. The fuzzy implementation in URV manipulator as a prototype system for marine application with vision and motion based controls are also available in^52^. Additionally, machine learning based approaches are also identified for designing and trajectory control of URV controller. The deep reinforcement learning with improved convolution network is employed for trajectory tracking of URV system in^53^ by Chu et al. The deep learning approaches implemented for surface-water robotic vehicle are discussed in^32^ in detail. In^32^, Qiao et al. presented a survey on deep learning approaches implemented for surface-water type robotic vehicles used in marine application. The machine learning approaches presented in^32^ can be further utilized for better control design of URV systems over other classical control strategies. Some of the researchers provided the significant importance to control strategies on vision based controllers for URV systems. In^54^, vision based sensing system is implemented with light weight object detector embedded system for URV application by Wang et al. Moreover, specially designed URV prototype system named as Shark is developed in^55^ by Hong et al. by incorporating vision based controller for inspection system along with $H_\infty$ controller for URV body control.

Thus, the better URV control design can be proposed by combining the suitable control design approaches as discussed previously for trajectory tracking, speed and motion control, heave controller, vision based control, etc. In the next section, mathematical modelling of URV controller is done and presented in details.

## Mathematical modelling of underwater robotic vehicle controller

**Fig. 1:**
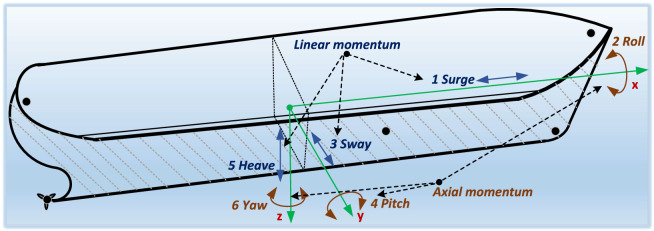
Dynamics of underwater robotic vehicle.

The momentum of underwater robotic vehicle (URV) is controlled by different hydrodynamic forces working on URV body. The URV is affected by six degree-of-freedom. These are surge, roll, sway, pitch, heave, and yaw. These forces are working on URV body for controlling forward and backward linear momentum, rolling around axis, swinging motion, steepness, dive force, and yawing across depth, respectively. All the hydrodynamic forces with degree-of-freedom are demonstrated in Fig. 1.

The mathematical modelling of URV is done by considering earth as reference frame to evaluate the URV position and its momentum. The determination of position and its momentum is accomplished by incorporating six degree-of-freedom by considering reference frame as URV body. Let linear momentum, surge, sway and heave be *x*, *y* and *z*, respectively while axial momentum, roll, pitch and yaw are depicted as $x_a,$ $y_a$ and $z_a,$ respectively. Similarly, with respect to global frame, the main hydrodynamics working on URV body are surge rate, sway rate and heave rate as linear dynamics, these are denoted as $\Delta x,$ $\Delta y$ and $\Delta z,$ respectively. However, axial momentum with respect to global frame are roll rate, pitch rate and yaw rate. These axial momentum are respectively represented as $\Delta x_a,$ $\Delta y_a$ and $\Delta z_a.$ The hydrodynamic forces experienced by URV body with respect to global and local frames, the position vector $(\bar{\rho })$ and velocity vector $(\bar{\nu })$ are represented as follows

1 $$\begin{aligned} \bar{\rho } = [x, y, z, x_{a}, y_{a}, z_{a}]^{T} \end{aligned}$$

2 $$\begin{aligned} \bar{\nu } = [\Delta x, \Delta y, \Delta z, \Delta x_{a}, \Delta y_{a}, \Delta z_{a}]^{T} \end{aligned}$$

Since, number of hydro-dynamical forces are applied in URV body, perturbation around URV body is accounted due to underwater stream current, surface effect, buoyancy effect, centre of gravity, cavitation effect, etc. These effects are main cause of perturbation in the normal trajectory of URV body^56^. The actuated and unactuated URV dynamics in the form of spatial transformation matrix (*J*) with respect to reference frame can be given as

3 $$\begin{aligned} \dot{\rho } = J(\bar{\rho }) \cdot \bar{\nu } \end{aligned}$$

In terms of actuated $(J_A)$ and unactuated $(J_U)$ dynamics, spatial transformation matrix can be rewritten as

4 $$\begin{aligned} \dot{\rho } = J_A(\bar{\rho }).\bar{\nu }_A+J_U(\bar{\rho })\cdot \bar{\nu }_U \end{aligned}$$

The mathematical representation of forces operating on URV body and their transformed matrices can be given as

5 $$\begin{aligned} {\bar{\tau}_h}+\bar{E}_d(t) = M(\bar{\nu })\dot{\nu }+C(\bar{\nu })\dot{\nu }+D(\bar{\nu })\dot{\nu }+B(\bar{\rho }) \end{aligned}$$

where, ${\bar{\tau }_h}$ and $\bar{E}_d(t)$ are hydro-dynamical control force and external disturbance vector, respectively. The inertia matrix for URV body and coriolis-centripetal matrix are represented by $M(\bar{\nu })$ and $C(\bar{\nu }),$ respectively. The $D(\bar{\nu })$ is depicting damping hydro-dynamical matrix. The effect of buoyancy forces and gravitational vectors are represented by $B(\bar{\rho })$ to incorporate counter balance dynamics. In the similar way, with the help of external and internal dynamics to calculate the URV trajectory^5^, the velocity transformation mapping can be expressed as

6 $$\begin{aligned} ({{\bar{\tau }_h}})_{\rho }+(\bar{E}_d(t))_\rho = M_{\rho }(\bar{\rho })\ddot{\rho }+C_{\rho }(\bar{\nu },\bar{\rho })\dot{\rho }+D_{\rho }(\bar{\nu },\bar{\rho })\dot{\rho }+B_{\rho }(\bar{\rho }) \end{aligned}$$

In URV body, the overall external forces are exerted by different dynamics. These dynamics affect the state variables of URV body. In the depiction of state variables, some assumptions are considered such as forward speed of URV body is taken constant, and pitch rate of URV body is considered as negligible^57^. Thus, the displacement in depth of URV body can be calculated as

7 $$\begin{aligned} \begin{array}{cc} \dot{y}_a = \Delta {y}_a\\ \dot{z}=-\Delta {x}\sin {y_a}+\Delta {z}\cos {y_a}\\ \text {i.e. } \dot{z \ \ } {\approx }-\Delta {x}+\Delta {z} \end{array}\hspace{1cm} \end{aligned}$$

By incorporating overall dynamics, the state space representation of URV system can be given as

8 $$\begin{aligned} \left. \begin{array}{cc} \dot{x} & = \bar{A}x+\bar{B}u \\ y & = \bar{C}x+\bar{D}u \end{array}\hspace{1cm}\right. \end{aligned}$$

In (8), for *x* state variables, the relation between input variables *u* and output variables *y* is presented by incorporating the URV system dynamics. In (8), $\bar{A}$ and $\bar{B}$ are state matrix of URV system and input matrix of URV system, respectively. While, output matrix and feed forward matrix of URV system are represented by $\bar{C}$ and $\bar{D},$ respectively, for depicting URV dynamics. The linearization of modelled URV system is done to obtain the stable system and controller can be designed easily. After linearization^58^, the third order URV controller is designed by considering depth with respect to stern displacement of URV. By considering state matrices with nominal controller gain and appropriate time lag for URV controller, the state space model of URV controller is obtained. By utilizing the numerical values of URV dynamics^8^ in (8), for state matrix, input matrix, output matrix and feed forward matrix, modelling of URV is accomplished as

9 $$\begin{aligned} \bar{A}= \begin{bmatrix} -3.7838 & -54.6340 & -28.8957 \\ \vspace{2mm} 1 & 0 & 0 \\ \vspace{2mm} 0 & 1 & 0 \end{bmatrix} \end{aligned}$$

10 $$\begin{aligned} {\bar{B}^{T} = \begin{bmatrix} 1&0&0 \end{bmatrix}} \end{aligned}$$

11 $$\begin{aligned} {\bar{C} = \begin{bmatrix} 0.9623&17.2990&15.3648 \end{bmatrix}} \end{aligned}$$

12 $$\begin{aligned} {\bar{D} = \begin{bmatrix} 0 \end{bmatrix}} \end{aligned}$$

The transfer function of URV controller represented as $\bar{W}_3(s)$ is obtained using (9)–(12) as

13 $$\begin{aligned} \bar{W}_3(s)=\frac{\bar{U}_3(s)}{\bar{V}_3(s)}=\left( \frac{0.9623s^{2}+17.299s+15.3648}{s^{3}+3.7838s^{2}+54.634s+28.8957}\right) \end{aligned}$$

where, *s* is the Laplace operator, $\bar{U}_3(s)$ denotes the numerator of HOURV controller in terms of depth, and $\bar{V}_3(s)$ is depicting the denominator of HOURV controller in terms of stern displacement of URV. The presented URV controller transfer function is of order three. This third order URV controller is further utilized for approximation in section “Numerical solution: approximation of URV controller, results and discussion”. In the next section, the problem formulation is presented to depict the reduction process.

## Problem formulation

### Depiction of Higher-order (HO) system and lower-order (LO) model

Let higher-order (HO) system of $h^{th}$ order be

14 $$\begin{aligned} \bar{W}_{h}(s)=\frac{\bar{U}_{i}(s)}{\bar{V}_{i}(s)}=\frac{\bar{U}_{0}+\bar{U}_{1}s+\bar{U}_{2}s^{2}+\cdots +\bar{U}_{h-1}s^{h-1}}{\bar{V}_{0}+\bar{V}_{1}s+\bar{V}_{2}s^{2}+\cdots +\bar{V}_{h}s^{h}} \end{aligned}$$

where, numerator terms $\bar{U}_i(s)$ are defined for *i* $\in$ $0,1,2,\cdots ,(h-1),$ while $\bar{V}_i(s)$ are denominator terms which are defined for *i* $\in$ $0,1,2,\cdots ,h.$

Suppose, the lower-order (LO) model for HO system (14) is reduced to order *r* such that $h>r.$ Let LO model be

15 $$\begin{aligned} {W^*_r}(s)=\frac{{U}^*_i(s)}{{V}^*_i(s)}=\frac{{U}^*_0+{U}^*_{1}s+{U}^*_{2}s^2+\cdots +{U}^*_{r-1}s^{r-1}}{{V}^*_0+{V}^*_{1}s+{V}^*_{2}s^2+\cdots +{V}^*_{r}s^r} \end{aligned}$$

where, ${U^*_i}(s)$ are the numerator terms for *i* $\in$ $0,1,2,\cdots ,(r-1)$ and ${V^*_i}(s)$ are denominator terms for *i* $\in$ $0,1,2,\cdots ,r.$

### Multi-point matching of expansion parameters of HO system and LO model

The expansion parameters of HO system and its LO model are obtained by matching around considered multi-points. Suppose, *n* number of multi-points are considered for matching to obtain the desired LO model of order *r*, where *n* is such that *n* $\le$ $(h+r-1)$ for better matching.

Let the multi-points be $n_1,n_2,n_3,\cdots ,n_{(h+r-1)}.$ Thus, specified points of matching are obtained as

16 $$\begin{aligned} N(s)=(s-n_{1})(s-n_{2})(s-n_{3})\cdots (s-n_{(h+r-1)}) \end{aligned}$$

The multi-point matching can be rewritten as

17 $$\begin{aligned} N(s)=\prod _{i=1}^{(h+r-1)}(s-n_{i}) \end{aligned}$$

The matching with considered multi-points is done by obtaining factorized augmented transfer functions (FATFs) as *X*(*s*) and *Y*(*s*) using (14) and (15). The ${(h+r-1)^{th}}$ order FATFs, *X*(*s*) and *Y*(*s*) are expressed as

18 $$\begin{aligned} X(s)=\bar{V}_i(s) \cdot {U^*_i}(s) \end{aligned}$$

19 $$\begin{aligned} Y(s)=\bar{U}_i(s) \cdot {V^*_i}(s) \end{aligned}$$

Now, equating the (18) and (19) with respect to (17) around expansion parameters. The errors between expansions parameters of (18) with respect to (17), and (19) with respect to (17) are desired to be minimized for obtaining the unknown LO model coefficients. The expansion parameters by considering desired multi-points are obtained as

20 $$\begin{aligned} X(s)_{new}=x_{0}+x_{1}s+x_{2}s^{2}+\cdots +x_{(h+r-1)}s^{(h+r-1)} \end{aligned}$$

21 $$\begin{aligned} Y(s)_{new}=y_{0}+y_{1}s+y_{2}s^{2}+\cdots +y_{(h+r-1)}s^{(h+r-1)} \end{aligned}$$

### Formulation of objective function and its constraints

For determination of unknown LO model coefficients, it is desired to minimize the error between (20) and (21). However, the objective function (OF) is framed by utilizing Routh equivalent coefficients of (20) and (21) with respect to (16). In the OF, ${(h+r-1)^{th}}$ terms are employed for determination of unknown coefficients of desired model depicted in (15). In this determination, minimization of error between (20) and (21) up to ${(h+r-1)^{th}}$ terms, is accomplished by minimizing the OF framed by utilizing the coefficients of (20) and (21). So the OF can be constructed^59^ as

22 $$\begin{aligned} J=w_{i}\sum _{i=1}^{(h+r-1)}{{\left[ \left( 1-\frac{X_i(s)_{new}}{Y_i(s)_{new}}\right) \right] }^{2}}_{around \ [s=n_{1},n_{2},\cdots ,n_{(h+r-1)}]} \end{aligned}$$

where, $w_{i}$ are the weights associated with the OF. The OF shown in (22) can be reconstructed as

23 $$\begin{aligned} J=w_{1}{{\left[ \left( 1-\frac{X_1(s)_{new}}{Y_1(s)_{new}}\right) \right] }^{2}}+w_{2}{{\left[ \left( 1-\frac{X_2(s)_{new}}{Y_2(s)_{new}}\right) \right] }^{2}}+ \cdots +w_{(h+r-1)}{{\left[ \left( 1-\frac{X_{(h+r-1)}(s)_{new}}{Y_{(h+r-1)}(s)_{new}}\right) \right] }^{2}} \end{aligned}$$

In the OF framed in (23), the weights are also associated to provide proper importance to each sub-objective based on the effect of multi-points on steady-state (SS) and transient state characteristics of LO model with respect to HO system. The values of associated weights are provided with equal importance of each sub-objectives. After assigning the weights, the desired LO model’s coefficients are manifested by minimizing the fitness function formulated in (22). In this process of minimization, satisfying the SS matching, and ensuring the Hurwitz criterion of stability (HCS) are considered as two prime constraints. These constraints are respectively depicted in (24) and (25).

24 $$\begin{aligned} \frac{\bar{U}_0(s)}{\bar{V}_0(s)}=\frac{{U}^*_0(s)}{{V}^*_0(s)} \end{aligned}$$

25 $$\begin{aligned} {{V}^*_i(s)} \text { of } (15), \text { must be Hurwitz.} \end{aligned}$$

The matching of SS is incorporated to ensure the zero SS error as given in (24). However, HCS as stated in (25), is providing stable LO model.

The associated weights with fitness-function shown in (22) are selected such that weights must be normally distributed among each sub-objectives. Thus, the resultant fitness-function is constructed by assigning the weights. The minimization of resultant fitness function is accomplished by incorporating the greywolf optimization algorithm (GWOA). The description of GWOA is presented in section “Greywolf optimization algorithm”.

## Greywolf optimization algorithm

Mirjalili et al. introduced the greywolf optimization algorithm (GWOA)^60^. GWOA is a population-based meta-heuristic algorithm inspired by social behavior and hunting strategy of grey wolves. The hierarchy followed by wolves in the pack is based on the domination behavior depicted by members of greywolf pack.

The GWOA begins with an initial population of wolves as candidate solutions. These are randomly distributed in the search space. The wolves’ positions are then iteratively updated to improve the quality of solutions. At each iteration, the algorithm simulates the social hierarchy within a wolf pack, consisting of an alpha, beta, and delta wolves. The alpha wolf represents the best solution found so far, while the beta and delta wolves correspond to the second-best and third-best solutions, respectively. The alpha wolf leads the hunting process by exploring promising regions in the search space. However, the beta and delta wolves perform local searches. The updated position of each wolf is determined by its proximity to the prey, which represents the optimal solution, as well as random exploration. Through a balance of exploration and exploitation, GWOA gradually converges towards an optimal or near-optimal solution. The GWOA terminates when a specified termination criterion is met, such as reaching a maximum number of iterations or achieving a desired level of solution quality. GWOA is applied to various optimization problems, and shows promising effect on obtaining effective and better solutions, efficiently. The pseudo code of GWOA is provided in Table 1.

The equations to represent the mathematical model for capturing the locating, encircling, and attacking processes involved in the hunting behavior of grey wolves can be given as

For locating the prey:

26 $$\begin{aligned} X_i(t+1) = X_ {prey}(t) - A .\bigg | C_i(t).\bigg (X_{{prey}}(t) - X_i(t)\bigg )\bigg | \end{aligned}$$

For encircling prey:

27 $$\begin{aligned} X_i(t+1) = X_{{prey}}(t) - A\cdot D_{i}(t) \end{aligned}$$

where, $$D_{i}(t)=\bigg | C_i(t) \cdot \bigg (X_{{prey}}(t) - X_i(t)\bigg )\bigg |$$

For attacking prey:

28 $$\begin{aligned} X_i(t+1) = \frac{1}{3} \bigg [ [X_{alpha}-A_{1}D_{alpha}]+ [X_{beta}-A_{1}D_{beta}] +[X_{delta}-A_{1}D_{delta}] \bigg ] \end{aligned}$$

29 $$\begin{aligned} X_i(t+1) = \frac{1}{3} \bigg [ X_{i\alpha }+ X_{i\beta } + X_{i\delta } \bigg ] \end{aligned}$$

where, $t$ represents the current iteration, $A$ is the coefficient for exploration following the negative slope of [2, 0] such that $A=2.\bar{\psi }.\bar{\phi }-\bar{\psi },$ for $\bar{\psi }\in [2, 0]$ and $\bar{\phi }\in [0, 1].$ The coefficient $C_i(t)$ is such that $C_i(t)=2.\bar{\theta }$ for $\bar{\theta }$ $\in$ [0, 1]. Further, $X_i(t)$ is the position of the $i$-th wolf, $X_{{prey}}(t)$ is the position of the prey, $X_{{alpha}}(t)$ is the position of the alpha wolf, $D_{{alpha}}(t)$ is a random vector in the range [0, 1]. Similar definition can be considered for other wolves i.e. $X_{beta}$ and $X_{delta}.$ Furthermore, $D_{{prey}}(t)$ is a random vector defined for the position of prey in the range [0, 1].

**Table 1:**
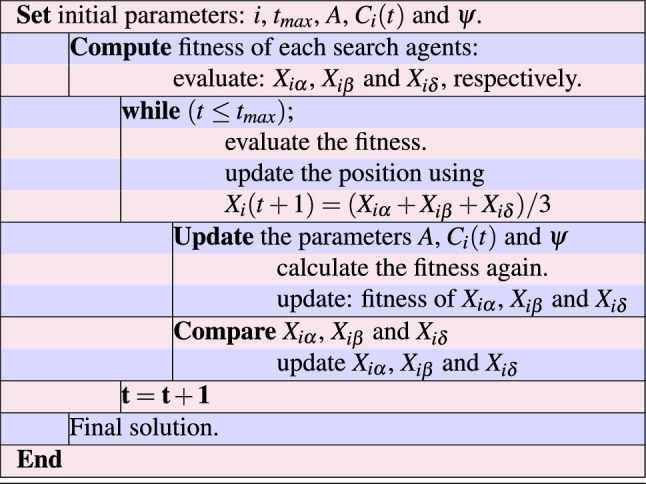
Pseudo code of GWO algorithm.

## Numerical solution: approximation of URV controller, results and discussion

The underwater robotic vehicle (URV) controller^8^ of order three as depicted in (13) of section “Mathematical modelling of underwater robotic vehicle controller” is lowered to model of order two. The transfer-function of $3^{rd}$ order URV controller system is

30 $$\begin{aligned} \bar{W}_3(s)=\frac{\bar{U}(s)}{\bar{V}(s)}=\left( \frac{0.9623s^{2}+17.299s+15.3648}{s^{3}+3.7838s^{2}+54.634s+28.8957}\right) \end{aligned}$$

The system of URV controller shown in (30) is desired to approximate to lower-order (LO) model of order two. Let LOURV model be

31 $$\begin{aligned} {W^*_2}(s)=\frac{{U}^*(s)}{{V}^*(s)}=\frac{{U}^*_0+{U}^*_{1}s}{{V}^*_0+{V}^*_{1}s+s^2} \end{aligned}$$

The determination of desired LOURV model is done with the help of multi-point matching. Since, the order of HOURV is 3, and desired LOURV model’s order is 2, the required number of points to be matched as multi-point matching must be $(3+2)-1=4.$ The four matching points are considered as $s=0,1,2,3.$

The selection of multiple matching points i.e. $s={0,1,2,3}$ corresponds to improved response of LOURV model with respect to HOURV system. These multi-points are carefully chosen expansion points that are strategically placed to capture essential dynamic characteristics of the HOURV system across a wide range of frequency. In which, specifically selected point i.e., $s=0$ corresponds to steady-state (SS) time, which directly maps to the SS behavior of the LOURV model for HOURV system. This ensures that the LOURV model accurately retains the long-term equilibrium response of the HOURV system. Moreover, multi-points $s=1,2,3$ represent increasing locations in the complex frequency domain that correspond inversely to specific time points in the time domain. By matching around aforementioned points, the evaluations of the HOURV system response at transient time can be done in obtaining better LOURV model. The multi-points $s=1,2,3$ are crucial in accurately approximating fast dynamics, overshoots, and settling behaviors of the HOURV system. The inclusion of selected matching points ensures that the desired LOURV model maintains fidelity not only at steady state but also during critical transitional phases. Since, the point $s=0$ is intentionally considered to minimize the SS error between HOURV system and LOURV model which is further utilized as constraint. Thus, matching is done around $s=0,1,2,3$ by obtaining the characteristics equation as

32 $$\begin{aligned} N(s)=s(s-1)(s-2)(s-3)=s^4-6s^3+11s^2-6s \end{aligned}$$

The matching with considered multi-point is done by obtaining factorized augmented transfer functions (FATFs) as *X*(*s*) and *Y*(*s*) using (14) and (15). The FATFs *X*(*s*) and *Y*(*s*) are expressed as

33 $$\begin{aligned} X(s)=\bar{V}(s) \cdot {U^*}(s) \end{aligned}$$

34 $$\begin{aligned} Y(s)=\bar{U}(s) \cdot {V^*}(s) \end{aligned}$$

With the help of numerator and denominator coefficients of (30) and (31), the FATFs become

35 $$\begin{aligned}  {{X}(s)}_{new}={U}^*_{1}s^{4}+(3.7838{U}^*_{1}+{U}^*_{0})s^{3}+  (54.634{U}^*_{1}+3.7838{U}^*_{0})s^{2}+ (28.8957{U}^*_{1}+54.634{U}^*_{0})s+ 28.8957{U}^*_{0} \end{aligned}$$

36 $$\begin{aligned}  {Y(s)}_{new}=0.9623s^{4}+(0.9623{V}^*_{1}+17.299)s^{3}+(17.299{V}^*_{1}+0.9623{V}^*_{0}+15.3648)s^{2}+(15.3648{V}^*_{1}+17.299{V}^*_{0})s+15.36487{V}^*_{0} \end{aligned}$$

Now, equating the (35) and (36) with respect to (32) around expansion parameters i.e. $s=0$ to $(h+r-1)=4.$ Among these points, matching around $s=0$ is necessarily done. The matching around $s=0$ is considered as one of the constraint in the minimization of objective function (OF). The OF expressed in (22) turns out to be

37 $$\begin{aligned} J=w_{i}\sum _{i=1}^{4}{{\left[ \left( 1-\frac{X(s)_{new}}{Y(s)_{new}}\right) \right] }^{2}}_{around \ [s=0,1,2,3]} \end{aligned}$$

The minimization of errors between FATFs derived in (35) and (36) are accomplished by minimizing the OF framed in (37). Thus, the OF can be rewritten as

38 $$\begin{aligned}  &J=w_{1}{{\left[ \left( 1-\frac{28.895{U}^*_{0}}{15.3648{V}^*_{0}}\right) \right] }^{2}}+ w_{2}{{\left[ \left( 1-\frac{34.8957{U}^*_{1}+54.634{U}^*_{0}}{15.3648{V}^*_{1}+17.299{V}^*_{0}+5.7738}\right) \right] }^{2}}+ \\ & w_{3}{{\left[ \left( 1-\frac{43.634{U}^*_{1}+3.7838{U}^*_{0}}{17.299{V}^*_{1}+0.9623{V}^*_{0}+4.7795}\right) \right] }^{2}} +  \\ & w_{4}{{\left[ \left( 1-\frac{9.7838{U}^*_{1}+{U}^*_{0}}{0.9623{V}^*_{1}+23.0728}\right) \right] }^{2}} \end{aligned}$$

**Fig. 2:**
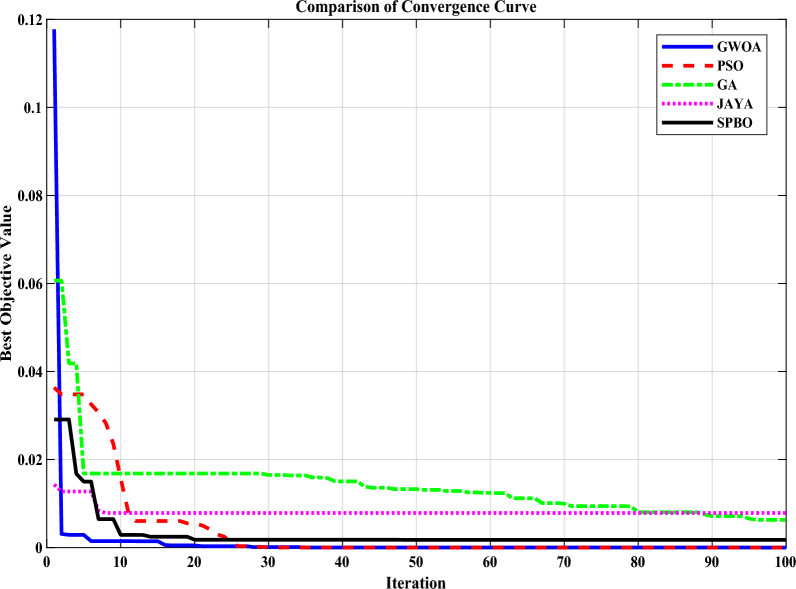
Comparison of Convergence Curve in Determination of LOURV model.

In (38), the associated weights $w_i$ for $i\in$ 1 to 4 are considered, such that the equal importance are provided to each sub-objectives. In this work, the weights were chosen uniformly to assign equal importance to each of the four sub-objectives. This decision was made to ensure a balanced trade-off between transient response and SS response at the selected multi-points, thereby maintaining consistency in performance across the approximation of HOURV system in terms of LOURV model. The equal weighting strategy is specifically adopted to prevent bias towards any single performance criterion, and to evaluate the baseline effectiveness of the proposed approximation under uniform influence of each sub-objective. This approach aligns with prior studies where equal weights are used as a starting point for evaluating multi-objective behavior in the absence of clearly dominant criteria. Since the weights are equal, so importance provided to each sub-objective is uniformly considered, which indicates that there is moderate sensitivity among each sub-objective. The weight calculation constraint is given in (39).

39 $$\begin{aligned} \sum _{i=1}^{k=(h+r)-1}\Bigg {[}w_i^{k}\Bigg {]}=1 \end{aligned}$$

Thus, weighted distribution is done as $w_{1}=w_{2}=w_{3}=w_{4}=0.25.$ Hence, the weighted OF is obtained as

40 $$\begin{aligned}  &J=0.25{{\left[ \left( 1-\frac{28.895{U}^*_{0}}{15.3648{V}^*_{0}}\right) \right] }^{2}}+ \\ & 0.25{{\left[ \left( 1-\frac{34.8957{U}^*_{1}+54.634{U}^*_{0}}{15.3648{V}^*_{1}+17.299{V}^*_{0}+5.7738}\right) \right] }^{2}}+ \\ & 0.25{{\left[ \left( 1-\frac{43.634{U}^*_{1}+3.7838{U}^*_{0}}{17.299{V}^*_{1}+0.9623{V}^*_{0}+4.7795}\right) \right] }^{2}}+ \\ & 0.25{{\left[ \left( 1-\frac{9.7838{U}^*_{1}+{U}^*_{0}}{0.9623{V}^*_{1}+23.0728}\right) \right] }^{2}} \end{aligned}$$

**Fig. 3:**
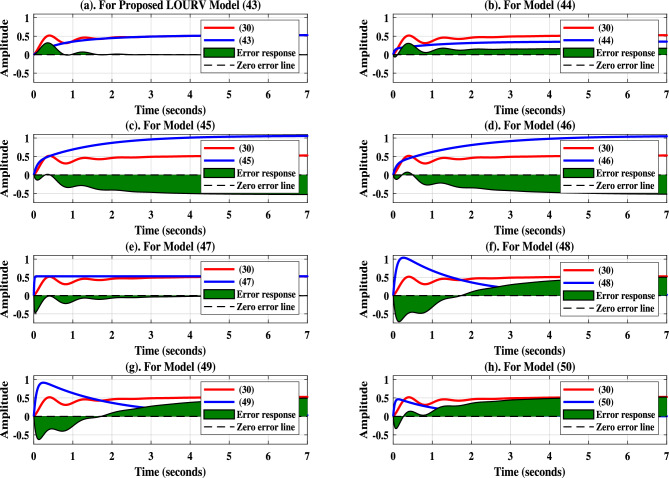
Step-response of HOURV controller and its LOURV model besides error response.

The greywolf optimization algorithm (GWOA) is chosen in the minimization of the framed OF under defined constraints, due to the advantages associated with GWOA in handling multi-objective, nonlinear optimization problems, particularly those involving conflicting criteria such as transient and steady-state accuracy. GWOA offers a good balance between exploration and exploitation by adapting its hierarchical leadership-based hunting behavior, which helps in effectively navigating the search space without premature convergence. This issue often encountered with algorithms like particle swarm optimization (PSO), genetic algorithm (GA), jaya optimization algorithm (JOA), student psychology-based optimization (SPBO), etc. The main highlights of GWOA are its simplicity, presence of fewer tunable parameters, ease of implementation in search space, also make it robust and computationally efficient for optimization problems. Furthermore, preliminary comparative simulations revealed that GWOA achieved faster convergence and produced lower-order models with improved fidelity as compared to PSO, GA, JOA, SPBO, for the same set of multi-point matching conditions. Its primary limitation is that, like other population-based algorithms, it may become computationally expensive for very high-dimensional spaces or suffer from stagnation in late iterations if diversity is not maintained. However, based on presented multi-objective problem structure and simulation outcomes, GWOA demonstrated consistent and reliable performance, justifying its use within the scope. In the support of GWOA, convergence curve is also provided in Fig. 2.

The weighted OF shown in (40) is minimized using greywolf optimization algorithm (GWOA) by satisfying zero SS error and Hurwitz criterion of stability (HCS) constraints as defined in (24) and (25), respectively. To solve (40), constraints (24) and (25) are changed to the constraints redefined in (41) and (42), respectively.

41 $$\begin{aligned} {{V}^*_0(s)}=1.8807{{U}^*_0(s)} \end{aligned}$$

42 $$\begin{aligned} \text {The denominator}, {{V}^*(s)} \hbox { of } (31), \hbox {must be Hurwitz, i.e. } {{V}^*_0.{V}^*_{1}>0}, \hbox { and } {{V}^*_0}>0, {{V}^*_{1}>0} \end{aligned}$$

In the minimization of OF, the GWOA parameters are selected for four decision variables with population size of 30, and the maximum number of iterations is taken as 100. The algorithm was terminated when attains its iteration limit or when the improvement in the objective function is obtained over 10 consecutive iterations. Additionally, the enforcement of critical system-theoretic constraints, particularly Hurwitz stability of LOURV model, was handled using a penalty function-based approach within the GWOA framework. During each candidate solution evaluation, the algorithm checked the eigenvalues of LOURV model’s. The simulation is performed on a standard computing platform equipped with an Intel Core i7 processor, 16 GB RAM, and MATLAB R2023a environment. By employing GWOA in (40) under constraints (41) and (42), the LOURV model is obtained as

43 $$\begin{aligned} {W}^*_2(s)=\frac{{U}^*(s)}{{V}^*(s)}=\frac{0.64831+0.697598s}{1.21924+2.609954{s}+{s^{2}}} \end{aligned}$$

**Table 2:** Specifications of time-domain (STD) of HOURV controller and its LOURV models

System and approximants	Specifications of time-domain (STD)				
	Rise time (s)	Settling time (s)	Peak (%)	Peak time (s)	Undershoot (%)
HOURV System (30)	0.2728	5.5463	0.5313	11.3237	0
Proposed model (43)	2.5830	5.2662	0.5307	9.1621	0
Model ${W}^*_{2(Diff)}(s)$ (44)	2.0939	4.0740	0.3544	9.4167	0
Model ${W}^*_{2(Diff-RA)}(s)$ (45)	2.9095	5.4173	1.0628	10.8675	0
Model ${W}^*_{2(Diff-DT)}(s)$ (46)	3.5601	6.5148	1.0628	12.9245	0
Model ${W}^*_{2(FD)}(s)$ (47)	0.0216	0.0399	0.5297	0.1021	0
Model ${W}^*_{2(FD-RA)}(s)$ (48)	0.0005651	6.3791	1.0383	0.2582	$1.057\times 10^{04}$
Model ${W}^*_{2(FD-DT)}(s)$ (49)	0.0005647	7.4312	0.9122	0.2454	$9.272\times 10^{03}$
Model ${W}^*_{2(FD-Diff)}(s)$ (50)	0.0001881	4.9579	0.4637	0.1313	$1.419\times 10^{04}$

**Fig. 4:**
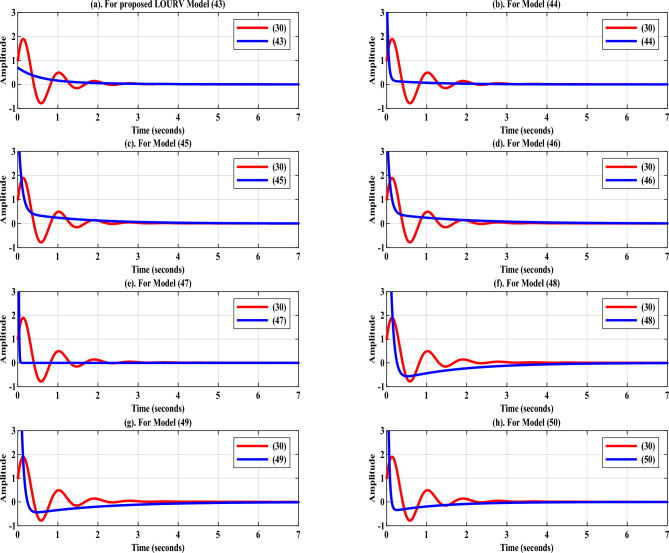
Impulse-response of HOURV controller and its LOURV model.

The superiority and efficacy of proposed LOURV model of order two as obtained in (43) is compared with the models ascertained by incorporating other approximation approaches such as differentiation (Diff) approximation, factor division (FD) approximation, and FD-Diff based mixed approximation. Along with these approximation approaches some other mixed approach of approximation are also utilized in inclusion with Diff and FD approximations as Diff-Routh approximation (Diff-RA), Diff-direct truncation (Diff-DT) approximation, FD-RA, FD-DT approximation.

The second order LOURV model obtained using Diff approximation is expressed in (44) as

44 $$\begin{aligned} {W}^*_{2(Diff)}(s)=\frac{30.7296+17.299s}{86.6871+109.2680{s}+3.7838{s^{2}}} \end{aligned}$$

Similarly, LOURV models of order two by employing Diff-RA and Diff-DT approaches are demonstrated in (45) and (46), respectively.

45 $$\begin{aligned} {W}^*_{2(Diff-RA)}(s)=\frac{30.7296+17.299s}{28.8957+46.9973{s}+3.7838{s^{2}}} \end{aligned}$$

46 $$\begin{aligned} {W}^*_{2(Diff-DT)}(s)=\frac{30.7296+17.299s}{28.8957+54.634{s}+3.7838{s^{2}}} \end{aligned}$$

The FD approximation approach provides the second order LOURV model as

47 $$\begin{aligned} {W}^*_{2(FD)}(s)=\frac{0.281233+54.7120s}{0.5289+103.2982{s}+{s^{2}}} \end{aligned}$$

Further, FD-RA and FD-DT approaches are implemented to obtain the second order LOURV models as depicted in (48) and (49), respectively.

48 $$\begin{aligned} {W}^*_{2(FD-RA)}(s)=\frac{0.281233+54.7120s}{28.8957+46.9973{s}+3.7838{s^{2}}} \end{aligned}$$

49 $$\begin{aligned} {W}^*_{2(FD-DT)}(s)=\frac{0.281233+54.7120s}{28.8957+54.634{s}+3.7838{s^{2}}} \end{aligned}$$

Furthermore, the FD-Diff based mixed approximation is employed to ascertained the LOURV model as provided in (50).

50 $$\begin{aligned} {W}^*_{2(FD-Diff)}(s)=\frac{0.281233+54.7120s}{86.6871+109.2680{s}+3.7838{s^{2}}} \end{aligned}$$

**Fig. 5:**
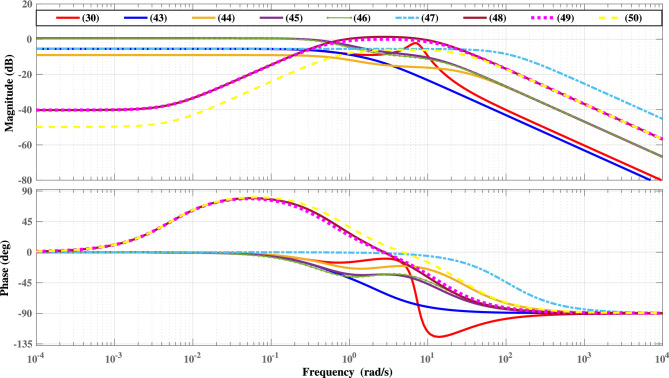
Bode-response of HOURV controller and its LOURV model.

**Table 3:** Performance error values (PEVs) of HOURV controller and its LOURV models

HOURV System (30) vs LOURV models	Performance Error Values (PEVs)					
	IAE	ISE	ITAE	ITSE	IT$^2$AE	IT$^2$SE
Proposed Model (43)	**0.180903**	**0.034234**	**0.152121**	**0.014230**	**0.403700**	**0.008130**
Model ${W}^*_{2(Diff)}(s)$ (44)	1.624736	0.274808	8.454500	1.437018	57.429889	9.897093
Model ${W}^*_{2(Diff-RA)}(s)$ (45)	4.598561	2.262194	25.645818	13.256049	174.959655	91.852348
Model ${W}^*_{2(Diff-DT)}(s)$ (46)	4.345791	2.057186	24.872123	12.544000	171.583581	88.526922
Model ${W}^*_{2(FD)}(s)$ (47)	0.380950	0.056639	0.628002	0.040192	1.939324	0.068294
Model ${W}^*_{2(FD-RA)}(s)$ (48)	4.095645	1.895552	23.053487	11.132869	163.306272	81.224230
Model ${W}^*_{2(FD-DT)}(s)$ (49)	3.883530	1.724812	22.484244	10.655955	160.447493	78.625718
Model ${W}^*_{2(FD-Diff)}(s)$ (50)	4.358950	2.082206	24.960694	12.658655	172.057285	89.006414

**Table 4:** Statistical analysis in the support of proposed LOURV model considering PEVs.

Statistic	IAE	ISE	ITAE	ITSE	IT$^2$AE	IT$^2$SE
F-statistic	25.664	17.470	24.177	17.260	23.709	17.370
p-value	0.00028	0.00128	0.00036	0.00134	0.00039	0.00131
Significance level $(\le 0.05)$	Significant					

In the support of proposed second order LOURV model as obtained in (43) for HOURV system shown in (30), step-response accompanying impulse and Bode-responses are presented. The suitability of proposed LOURV model manifested in (43) over other models depicted in (44)–(50) for HOURV system is defended by providing the error-response. The step and error-responses of LOURV models and HOURV system are provided in Fig. 3. In Fig. 3a, it can be clearly observed that the response of proposed LOURV model is close to the response of HOURV system in comparison to other LOURV models of (44)–(50). The error-response depicted in Fig. 3a for proposed LOURV model (43) with respect to HOURV system (30) is comparatively better as compare to error responses plotted in Fig. 3b–h for models (44)–(50), respectively. This proves that the error-response of proposed LOURV model (43) is comparatively better and acceptable with respect to HOURV system (30).

Similar observation is also found in Fig. 4 for proposed LOURV model (43) with respect to HOURV system (30). The impulse-response provided in Fig. 4a for proposed LOURV model (43) shows better representation in comparison with the responses plotted in Fig. 4b–h. In addition to this, Bode-response is also provided for further analysis of proposed LOURV model (43) obtained for HOURV system (30) in Fig. 5. In Fig. 5, the magnitude and phase responses of proposed LOURV model (43) are closely matching the magnitude and phase responses of the HOURV system expressed in (30) in comparison with the models obtained in (44)–(50). Thus, the impulse and Bode-responses as depicted in Figs. 4 and 5, respectively, are representing the better depiction of transient and frequency responses in the support of proposed LOURV model with respect to HOURV system (30). In these responses also, the characteristics of proposed LOURV model is comparatively suitable for HOURV system in comparison to models of (44)–(50).

Additionally, the specifications of time-domain (STD) and performance error values (PEVs) of proposed LOURV model (43) and other LOURV models as presented in (44)–(50) with respect to HOURV system are also provided in tabulated form for comparative analysis, and better explanation of proposed LOURV model. The STD are shown in Table 2 whereas Table 3 is providing the information about PEVs. Table 2 is provided in the support of presented step responses which provides numerical values of all STD to prove the efficacy of proposed LOURV model. Similar observation is found in Table 3, since the PEVs of proposed LOURV model is comparatively superior over other models of (44)–(50). In the support of the PEVs of the proposed LOURV model, analysis of variance for PEVs is done. In this statistical test, F-statistic and p-value are calculated to determine the significance level. A higher F-statistic indicates significant variance within the groups. Similarly, p-value signifies the probability of observing the null hypothesis. The p-value, typically less than 0.05 is indicating in the support of strong statistical significance by rejecting the null hypothesis. Since, the F-statistic and p-value as presented in Table 4, are statistically significant. In Table 4, it can be observed that F-statistic range from 17.260 to 25.664, which are considerably high for all PEVs. Moreover, PEVs yielded low p-values $(\le 0.00134).$ Thus, statistical data confirm that the proposed LOURV model (43) exhibits statistically significant improvement for respective HOURV system (30).

**Fig. 6:**
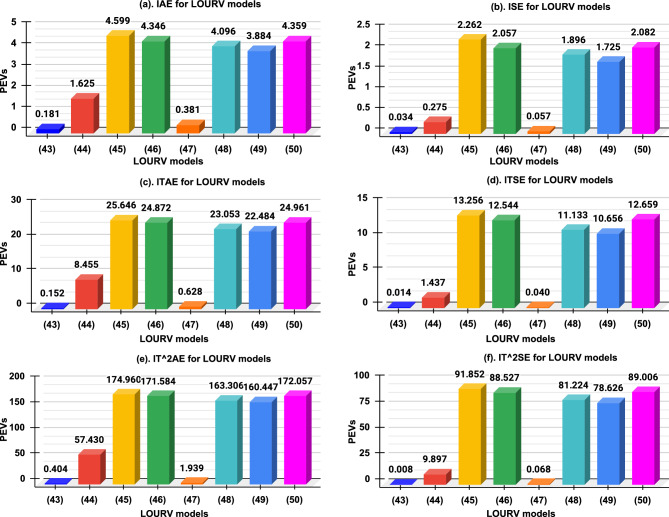
Performance error value plot of LOURV models with respect to HOURV system.

The graphical depiction of PEVs of proposed LOURV model and other LOURV models are also deployed in terms of bar plot in Fig. 6. In Fig. 6a–f, it is visible that the proposed model (43) is holding the minimum value of PEVs in comparison to LOURV models provided in (44)–(50). From Table 3 and Fig. 6, it can be observed that the proposed LOURV model as obtained in (43), holds the minimum PEVs in comparison with other LOURV models presented in (44)–(50) with respect to HOURV system.

Hence, it can be concluded from responses and comparative analysis of tables’ data that the proposed LOURV model (43) is depicting the similar properties of HOURV system (30) which also prove the adequacy of proposed LOURV model.

## Conclusion

This presented research is providing approximated lower-order underwater robotic vehicle (LOURV) model for higher-order (HO) URV controller. The multi-point matching technique is utilized in this approximation by employing greywolf optimization algorithm (GWOA). The LOURV model is obtained by matching the expansion parameters of HOURV controller and its desired LOURV controller around multi-points. For selected multi-points, matching is done by exploiting factorized augmented transfer functions, then the errors between expansion parameters are minimized. The error minimization is done by obtaining the optimized solution for constructed objective function (OF). The proposed LOURV model is supported by responses and tabular comparative analysis. From presented results, it can be concluded that the proposed LOURV model suits well for HOURV controller. In future, associated weights depicted in OF can be ascertained by implementing suitable systematic approach. The detailed future scope are given as follows:

- Due to higher degree of freedom, approximation approaches can be implemented for other robotic applications such as surface robot including linear momentum based and axial momentum based, aerial robot, unmanned robotic vehicles, floating robotic vehicle, etc.
- In the field of bio-medical engineering, various bio-medical instruments can be design efficiently by incorporating appropriate approximation technique.
- Some of the work on approximations can be also exploited for micro-electronics mechanical systems (MEMSs) for providing economical circuit design.
- The systematic learning process such as analytic hierarchy process, simple-additive-weighting method, R-method, multi-attribute utility and value theory, TOPSIS (Technique for Order of Preference by Similarity to Ideal Solution) method, graph-theory, grey-relational analysis, etc., can be implemented for obtaining better approximated model.
- Additionally, multi-point matching can be implemented with weight determination approach by implementing learning based approach.

Further, control design can also be done by employing different control design methods by incorporating the machine learning approach. By including the possibility of integrating the proposed LOURV controller with embedded platforms or real-time control hardware, in future work, computational feasibility and validation can be done under hardware-in-the-loop (HIL) testing scenarios. Furthermore, as a future work direction, the uncertainty in HOURV system due to variation in system dynamics can be addressed to propose an interval modelled URV system for detailed analysis of system and its controller.

## Data Availability

The data used and/or analyzed during the current study are available from the corresponding author upon reasonable request.

## Abbreviations

COBOT: Collaborative-robot
Diff: Differentiation
DT: Direct truncation
FATF: Factorized augmented transfer function
FD: Factor division
GWOA: Greywolf optimization algorithm
HCS: Hurwitz criterion of stability
HIL: Hardware-in-the-loop
HO: Higher-order
IO-1: Intelligent ocean-1
LO: Lower-order
MEMS: Micro-electronics mechanical system
NMPCS: Nonlinear model predictive control scheme
OF: Objective function
PEV: Performance error value
RA: Routh approximation
SS: Steady-state
TOPSIS: Technique for order of preference by similarity to ideal solution
TWINBOT: Twin robot
URV: Underwater robotic vehicle

## References

[1] Hadi, B., Khosravi, A. & Sarhadi, P. Adaptive formation motion planning and control of autonomous underwater vehicles using deep reinforcement learning. *IEEE J. Ocean. Eng.* (2023).

[2] Luvisutto, A., Celani, A., Renda, F., Stefanini, C. & De Masi, G. Enhancing collaboration in uncertain environment: Multi-agent reinforcement learning for underwater monitoring. *Exp. Syst. Appl.* 127256 (2025).

[3] Sahoo, A., Dwivedy, S. K. & Robi, P. Advancements in the field of autonomous underwater vehicle. *Ocean Eng.* **181**, 145–160 (2019).

[4] Hasan, K. *et al.* Oceanic challenges to technological solutions: A review of autonomous underwater vehicle path technologies in biomimicry, control, navigation and sensing. *IEEE Access* (2024).

[5] Wu, Y., Low, K. H. & Lv, C. Cooperative path planning for heterogeneous unmanned vehicles in a search-and-track mission aiming at an underwater target. *IEEE Trans. Veh. Technol.* **69**, 6782–6787 (2020).

[6] Ratajczak, J. & Tchoń, K. Normal forms and singularities of non-holonomic robotic systems: A study of free-floating space robots. *Syst. Control Lett.* **138**, 104661 (2020).

[7] Ciuccoli, N., Screpanti, L. & Scaradozzi, D. Underwater simulators analysis for digital twinning. *IEEE Access* (2024).

[8] Kadam, S. D. & Tiwari, K. N. A simplified approach to tune pd controller for depth control of an autonomous underwater vehicle. In *Proceedings of National conference on Communication, Computing and Networking Technologies*, pp. 209–212 (2013).

[9] Yadav, U. K. & Singh, V. R-method-based reduction of continuous systems using grey wolf optimization algorithm. *Circ. Syst. Signal Process.* pp. 1–30 (2022).

[10] Yang, G., Wang, H., Yao, J. & Zou, X. Multilayer neurocontrol of servo electromechanical systems with disturbance compensation. *Appl. Soft Comput.* **151**, 111043 (2024).

[11] Yang, G. & Yao, J. Multilayer neurocontrol of high-order uncertain nonlinear systems with active disturbance rejection. *Int. J. Robust Nonlinear Control* **34**, 2972–2987 (2024).

[12] Yang, G. State filtered disturbance rejection control. *Nonlinear Dyn.* **113**, 6739–6755 (2025).

[13] Mendonça, G., Afonso, F. & Lau, F. Model order reduction in aerodynamics: Review and applications. *Proc. Inst. Mech. Eng. G J. Aerospace Eng.* **233**, 5816–5836 (2019).

[14] Yuan, C. et al. Towards efficient design optimization of a miniaturized thermoelectric generator for electrically active implants via model order reduction and submodeling technique. *Int. J. Numer. Methods Biomed. Eng.* **36**, e3311 (2020). 10.1002/cnm.3311 31943823

[15] Yadav, U. K., Monga, H., Meena, V., Mande, P. & Singh, V. Error-minimization oriented approximation of voltage-multiplier based dc-dc level-up higher-order converter. In *2022 IEEE International Conference on Power Electronics, Drives and Energy Systems (PEDES)*, pp. 1–5 (IEEE, 2022).

[16] Himpe, C., Grundel, S. & Benner, P. Model order reduction for gas and energy networks. *J. Math. Ind.* **11**, 1–46 (2021).33425640

[17] Yadav, U. K., Meena, V. & Singh, V. A novel rank-order-centroid based reduction of self-balanced-bicycle-robot controller using grey-wolf optimizer. *J. Intell. Robot. Syst.* **106**, 1–15 (2022).

[18] Lee, J. & Choi, S. B. Integrated control of steering and braking for path tracking using multi-point linearized mpc. *IEEE Trans. Intell. Veh.* (2022).

[19] Potturu, S. R. & Prasad, R. Model order reduction of lti interval systems using differentiation method based on kharitonov’s theorem. *IETE J. Res.* **68**, 2079–2095 (2022).

[20] Prajapati, A. K. & Prasad, R. Reduced-order modelling of LTI systems by using routh approximation and factor division methods. *Circ. Syst. Signal Process.* **38**, 3340–3355 (2019).

[21] Sikander, A. & Prasad, R. Linear time-invariant system reduction using a mixed methods approach. *Appl. Math. Model.* **39**, 4848–4858 (2015).

[22] Kang, S., Yu, J., Zhang, J. & Jin, Q. Development of multibody marine robots: A review. *IEEE Access* **8**, 21178–21195 (2020).

[23] Yang, Y., Xiao, Y. & Li, T. A survey of autonomous underwater vehicle formation: Performance, formation control, and communication capability. *IEEE Commun. Surv. Tutor.* **23**, 815–841 (2021).

[24] Neira, J. et al. Review on unmanned underwater robotics, structure designs, materials, sensors, actuators, and navigation control. *J. Robot.* **2021**, 1–26 (2021).

[25] He, Y., Wang, D. B. & Ali, Z. A. A review of different designs and control models of remotely operated underwater vehicle. *Meas. Control* **53**, 1561–1570 (2020).

[26] Pi, R., Cieślak, P., Ridao, P. & Sanz, P. J. Twinbot: Autonomous underwater cooperative transportation. *IEEE Access* **9**, 37668–37684 (2021).

[27] Taesi, C., Aggogeri, F. & Pellegrini, N. Cobot applications-recent advances and challenges. *Robotics* **12**, 79 (2023).

[28] Wang, R., Wang, S., Wang, Y., Cheng, L. & Tan, M. Development and motion control of biomimetic underwater robots: A survey. *IEEE Trans. Syst. Man Cybern. Syst.* **52**, 833–844 (2020).

[29] Connor, J., Champion, B. & Joordens, M. A. Current algorithms, communication methods and designs for underwater swarm robotics: A review. *IEEE Sens. J.* **21**, 153–169 (2020).

[30] Cong, Y., Gu, C., Zhang, T. & Gao, Y. Underwater robot sensing technology: A survey. *Fundament. Res.* **1**, 337–345 (2021).

[31] Hoeher, P. A., Sticklus, J. & Harlakin, A. Underwater optical wireless communications in swarm robotics: A tutorial. *IEEE Commun. Surv. Tutor.* **23**, 2630–2659 (2021).

[32] Qiao, Y. *et al.* Survey of deep learning for autonomous surface vehicles in marine environments. *IEEE Trans. Intell. Trans. Syst.* (2023).

[33] Wibisono, A., Piran, M. J., Song, H.-K. & Lee, B. M. A survey on unmanned underwater vehicles: Challenges, enabling technologies, and future research directions. *Sensors* **23**, 7321 (2023). 37687776 10.3390/s23177321 PMC10490491

[34] Hermawan, Y. A., Suryadarma, M. I. & Yulianto, T. Design configuration of horizontal motion control system for unmanned underwater vehicle. In *IOP Conference Series: Earth and Environmental Science*, vol. 1461, 012040 (IOP Publishing, 2025).

[35] Sánchez, P. J. B., Papaelias, M. & Márquez, F. P. G. Autonomous underwater vehicles: Instrumentation and measurements. *IEEE Instrum. Meas. Magaz.* **23**, 105–114 (2020).

[36] Shi, L., Zheng, R., Liu, M. & Zhang, S. Distributed circumnavigation control of autonomous underwater vehicles based on local information. *Syst. Control Lett.* **148**, 104873 (2021).

[37] Liu, L., Zhang, L., Pan, G. & Zhang, S. Robust yaw control of autonomous underwater vehicle based on fractional-order pid controller. *Ocean Eng.* **257**, 111493 (2022).

[38] Bejarbaneh, E. Y., Masoumnezhad, M., Armaghani, D. J. & Pham, B. T. Design of robust control based on linear matrix inequality and a novel hybrid pso search technique for autonomous underwater vehicle. *Appl. Ocean Res.* **101**, 102231 (2020).

[39] Guerrero, J., Torres, J., Creuze, V. & Chemori, A. Adaptive disturbance observer for trajectory tracking control of underwater vehicles. *Ocean Eng.* **200**, 107080 (2020).

[40] Manzanilla, A. et al. Super-twisting integral sliding mode control for trajectory tracking of an unmanned underwater vehicle. *Ocean Eng.* **234**, 109164 (2021).

[41] Liu, X., Zhang, M., Chu, Z. & Rogers, E. A sphere region tracking control scheme for underwater vehicles. *IEEE Trans. Veh. Technol.* (2023).

[42] Sun, P. & Boukerche, A. Modeling and analysis of coverage degree and target detection for autonomous underwater vehicle-based system. *IEEE Trans. Veh. Technol.* **67**, 9959–9971 (2018).

[43] Heshmati-Alamdari, S., Eqtami, A., Karras, G. C., Dimarogonas, D. V. & Kyriakopoulos, K. J. A self-triggered position based visual servoing model predictive control scheme for underwater robotic vehicles. *Machines* **8**, 33 (2020).

[44] Heshmati-Alamdari, S., Karras, G. C. & Kyriakopoulos, K. J. A predictive control approach for cooperative transportation by multiple underwater vehicle manipulator systems. *IEEE Trans. Control Syst. Technol.* **30**, 917–930 (2021).

[45] Wang, X. Active fault tolerant control for unmanned underwater vehicle with actuator fault and guaranteed transient performance. *IEEE Trans. Intell. Veh.* **6**, 470–479 (2020).

[46] Chen, M. & Zhu, D. Optimal time-consuming path planning for autonomous underwater vehicles based on a dynamic neural network model in ocean current environments. *IEEE Trans. Veh. Technol.* **69**, 14401–14412 (2020).

[47] Liu, X., Zhang, M. & Rogers, E. Trajectory tracking control for autonomous underwater vehicles based on fuzzy re-planning of a local desired trajectory. *IEEE Trans. Veh. Technol.* **68**, 11657–11667 (2019).

[48] Mei, H., Yang, K., Liu, Q. & Wang, K. 3d-trajectory and phase-shift design for ris-assisted UAV systems using deep reinforcement learning. *IEEE Trans. Veh. Technol.* **71**, 3020–3029 (2022).

[49] Kong, F., Guo, Y. & Lyu, W. Dynamics modeling and motion control of an new unmanned underwater vehicle. *IEEE Access* **8**, 30119–30126 (2020).

[50] Lakhekar, G. V., Waghmare, L. M. & Roy, R. G. Disturbance observer-based fuzzy adapted s-surface controller for spatial trajectory tracking of autonomous underwater vehicle. *IEEE Trans. Intell. Veh.* **4**, 622–636 (2019).

[51] Wang, R. *et al.* Adaptive trajectory tracking control with novel heading angle and velocity compensation for autonomous underwater vehicles. *IEEE Trans. Intell. Veh.* (2023).

[52] Cai, M. et al. Grasping marine products with hybrid-driven underwater vehicle-manipulator system. *IEEE Trans. Autom. Sci. Eng.* **17**, 1443–1454 (2020).

[53] Chu, Z., Wang, F., Lei, T. & Luo, C. Path planning based on deep reinforcement learning for autonomous underwater vehicles under ocean current disturbance. *IEEE Trans. Intell. Veh.* **8**, 108–120 (2022).

[54] Wang, Y. et al. Real-time underwater onboard vision sensing system for robotic gripping. *IEEE Trans. Instrum. Meas.* **70**, 1–11 (2020).33776080

[55] Hong, L., Wang, X., Zhang, D.-S. & Zhao, M., & Xu, H. (Development, control, and evaluation. IEEE Trans. Intell. Veh. Vision-based underwater inspection with portable autonomous underwater vehicle, 2023).

[56] Xu, F., Wang, H., Au, K. W. S., Chen, W. & Miao, Y. Underwater dynamic modeling for a cable-driven soft robot arm. *IEEE/ASME Trans. Mechatron.* **23**, 2726–2738 (2018).

[57] Gibson, S. B. & Stilwell, D. J. Hydrodynamic parameter estimation for autonomous underwater vehicles. *IEEE J. Oceanic Eng.* **45**, 385–394 (2018).

[58] Zhu, D. et al. A hybrid control strategy of 7000 m-human occupied vehicle tracking control. *IEEE Trans. Intell. Veh.* **5**, 251–264 (2019).

[59] Yadav, U. K., Singh, V., Fortuna, L. & Sahu, U. K. Smart based multi-point matching assisted approximation of renewable interconnected power system. *IEEE Access* (2025).

[60] Mirjalili, S., Mirjalili, S. M. & Lewis, A. Grey wolf optimizer. *Adv. Eng. Softw.* **69**, 46–61 (2014).

